# Salinity Effect on Soil Bacterial and Archaeal Diversity and Assembly in *Phragmites australis* Salt Marshes in the Qaidam Basin, China

**DOI:** 10.3390/microorganisms13061253

**Published:** 2025-05-29

**Authors:** Pengcheng Zhu, Yuhui Wang, Wenyi Sheng, Mingyang Yu, Wei Wei, Wenlong Sun, Jian Gao, Zhenwei Xu, Ming Cao, Yuzhi Wang, Lele Liu, Weihua Guo

**Affiliations:** 1 Qingdao Key Laboratory of Ecological Protection and Restoration, School of Life Sciences, Shandong University, Qingdao 266237, China; zpc353edu@163.com (P.Z.); shwy@sdu.edu.cn (W.S.); 19880556989@163.com (M.Y.); weiv@mail.sdu.edu.cn (W.W.); qingyuansanren@163.com (W.S.); wangyuzhi@sdu.edu.cn (Y.W.); 2 National Glycoengineering Research Center, Shandong University, Qingdao 266237, China; wangyuhui@sdu.edu.cn; 3 Key Laboratory of Intelligent Health Perception and Ecological Restoration of Rivers and Lakes, Ministry of Education, Hubei University of Technology, 28 Linan Road, Wuhan 430068, China; jgao13@hotmail.com; 4 Institute of Ecology, Key Laboratory for Earth Surface Processes of the Ministry of Education, College of Urban and Environmental Sciences, Peking University, Beijing 100871, China; sddyxzw@163.com; 5 Center for Ecological Dynamics in a Novel Biosphere (ECONOVO), Department of Biology, Aarhus University, Ny Munkegade 114, DK-8000 Aarhus, Denmark; 6 College of Biotechnology, Tianshui Normal University, Tianshui 741001, China; yezhongchangxian@gmail.com

**Keywords:** salt marshes, community assembly, soil salinity, stochastic processes, microbial interactions

## Abstract

Extreme environments foster phylogenetically diverse microorganisms and unique community assembly patterns. Plateau saline marsh lakes represent understudied extreme habitats characterized by dual stressors of high salinity and low temperature. Here, we analyzed the soil bacterial and archaeal diversity in three salt marshes of the Qaidam Basin on the Qinghai-Tibetan Plateau, China. While the bacterial and archaeal alpha diversity showed no significant differences among the three salt marshes, the community composition varied significantly. Notably, soil salinity (indicated by electric conductivity, EC) exerted opposing effects on microbial diversity—suppressing bacterial while promoting archaeal communities. Stochastic processes were the predominant mechanism for both bacterial and archaeal community assembly, where the weights were, in descending order, drift, homogeneous selection, and dispersal limitation. Network analysis revealed predominantly positive co-occurrence patterns within both bacterial and archaeal communities. We did not find a direct relationship between any bacterial or archaeal co-occurrence network properties and soil EC, but there was a significant correlation of network complexity to microbial diversity, which was influenced by EC. Our findings indicate distinct responses of bacterial and archaeal diversity to varying salinity levels, while the underlying assembly processes appear to be conserved in driving shifts in community diversity in plateau salt marsh wetlands.

## 1. Introduction

Saline lakes constitute vital components of inland aquatic ecosystems, with a global distribution covering nearly one-quarter of the world’s lake surface area [1]. In plateau basins, declining precipitation has driven the transformation of numerous freshwater lakes into saline lakes and salt marshes. These ecosystems are valuable natural assets, holding significant ecological and scientific importance, and they play a crucial role in both preserving species diversity and regulating biogeochemical cycles locally, regionally and globally [2,3]. As a result of global changes in precipitation, evaporation, and human activities including salt and mineral mining, increased salinization is becoming a serious problem in salt marshes, threatening environmental quality, ecological health, and ecosystem functioning [4,5]. Elevated salinity levels can profoundly influence microbial community assembly, vegetation succession, and biogeochemical processes [6,7]. Therefore, investigating salinity’s impact on microbial communities may yield valuable insights for saline soil bioremediation and vegetation restoration, while also informing climate change mitigation strategies.

Soil microbial communities represent particularly sensitive biological indicators of environmental change, with cascading effects on plant communities and ecosystem functioning [8,9]. Salinization frequently serves as a primary environmental determinant shaping soil microbial composition in these regions [10,11]. While high salinity typically suppresses bacterial metabolic activity and growth, selectively favoring halotolerant species and consequently reducing bacterial diversity, contradictory findings suggest this pattern may not hold across all environments [12,13]. Some studies have reported negligible effects or even positive correlations between salinity and bacterial diversity in specific habitats [14,15,16]. Moreover, the effect of salinity on the community composition and diversity of archaea, another important microbiota in wetland soils, has also been controversial [17]. These differential responses across micro-ecosystems can generate ripple effects influencing overall ecosystem functionality [18]. Elucidating the ecological relationships between soil microbial communities and salinity gradients in plateau salt marshes is therefore essential for developing targeted management strategies.

In addition to the diversity and composition of microbial communities, growing attention is being given to community assembly mechanisms and microbial interactions [18,19]. Currently, it is generally accepted that stochastic processes (e.g., drift, dispersal) and deterministic processes (e.g., environmental selection by salinity) collectively govern microbial community assembly, and their relative contributions vary substantially across different habitats [20,21,22]. Notably, even within the same environment, bacteria and archaea often exhibit distinct assembly mechanisms [20]. However, the mechanism of bacterial and archaeal community assembly in saline lake wetlands is still unclear, which is vital for understanding the formation of soil bacterial and fungal community distribution patterns [23,24]. Microbial communities are collections of complex individuals in which microbial interaction patterns also influence microbial community dynamics and ecosystem functions [25]. Ecologically stable and diverse microbial communities typically demonstrate enhanced stress resistance, while providing sustained ecological services [26,27,28]. Co-occurrence network analysis has emerged as a powerful tool for deciphering these intricate microbial interactions, having been successfully applied across diverse ecosystems [29]. Nevertheless, current knowledge regarding how bacterial and archaeal co-occurrence networks respond to environmental changes, especially to soil salinization, is far from adequate.

The Tibetan Plateau is the highest plateau region in the world, covering an area of 2.5 million km^2^, with an average altitude of 4500 m. This unique region hosts thousands of saline lake wetlands exhibiting broad natural salinity gradients, making it an ideal system for studying salinity–microbiome relationships [30]. The Qaidam Basin, characterized by its basin topography and relatively lower elevation (2600–3000 m), represents a major concentration of saline lakes on the plateau. Since different plant communities possess unique soil microbial compositions [12], a typical plant of the Tibetan Plateau, *Phragmites australis*, was chosen as the study objective. Due to unique reproductive characteristics and salt adaptation, *Phragmites australis* is widely distributed around freshwater and saline lakes on the Tibetan Plateau. In this study, we analyzed the bacterial and archaeal communities in three *P. australis* around saline lakes in the Qinghai-Tibetan Plateau using amplicon high-throughput sequencing, and constructed a co-occurrence network. The specific goals of this study were to (1) clarify the assembly mechanisms of bacterial and archaeal communities; (2) explore the impact of soil salinity on microbial composition and function; and (3) reveal the key factors affecting microbial co-occurrence networks. We hypothesized that the unique environment of the Qinghai-Tibetan Plateau saline lakes would shape distinct microbial communities and interaction patterns in *P. australis*, with archaea showing higher salt-tolerance adaptation than bacteria.

## 2. Materials and Methods

### 2.1. Soil Sampling and Physicochemical Analysis

**Figure 1:**
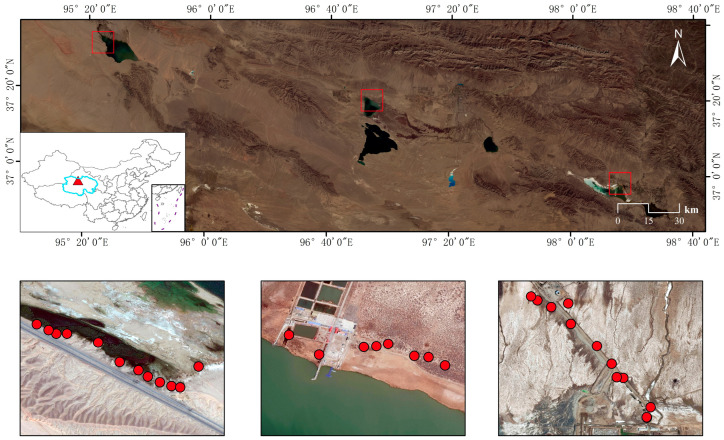
Geographical locations of soil samples around plateau saline lakes in the Qaidam Basin, China.

Qaidam Basin is located in northwestern China and is an important part of the Qinghai-Tibetan Plateau. There are many famous plateau saline lakes, where the common reed (*Phragmites australis*) is widely scattered. We collected topsoil samples (0–10 cm depth) from three distinct salt marshes associated with Keke Saline Lake (10 samples), Keluke Saline Lake (12 samples), and Xiaochaidan Saline Lake (10 samples) (Figure 1). At each sampling point, the surface layer of litter was gently disturbed and five subsamples were obtained using the five-point sampling method, and the subsamples were thoroughly mixed to represent one soil sample. After sampling, soil samples were preserved in ice boxes and sent back to the laboratory under preservation with dry ice. Subsequently, the soil samples were divided into two parts; one part was air-dried and used for measuring soil physicochemical properties, and the another part was stored in a −20 °C refrigerator for further DNA extraction.

Residual roots visible to the naked eye in the soil were removed and left to air dry at room temperature. Soil electric conductivity (EC) and pH were measured within soil–water extracts of 1:5 (*w*/*v*) using an electric conductivity meter and pH meter. Determination of soil total organic carbon (TOC) was carried out using the potassium dichromate method. After the soil samples had been fully decocted with concentrated sulfuric acid, soil total nitrogen (TN) and total phosphorus (TP) were determined using Kjeldahl nitrogen determination and molybdenum antimony colorimetry, respectively. Physicochemical properties of the soil at each sampling position are shown in Table S1.

### 2.2. DNA Extraction and Microbial Community Sequencing

Total soil DNA of each sample (approximate 0.20 g) was extracted using a DNeasy PowerSoil Kit (QIAGEN, Hilden, German), according to the instructions. The concentration and quality of DNA were detected using a NanoDrop^®^ ND-1000 spectrophotometer (Thermo Fisher Scientific Inc., Waltham, MA, USA) and 0.8% agarose gel electrophoresis. Bacterial communities and archaeal communities were sequenced by using the Illumina NovaSeq platform (OE Biotech Co., Ltd., Shanghai, China) on DNA samples that passed the test. Bacterial communities were sequenced by amplifying the V3V4 region of the 16S rRNA gene using primer 338F (3′-ACTCCTACGGGAGGCAGCA-5′) and 806R(3′-GGACTACHVGGGTWTCTAAT-5′), while archaeal communities were sequenced by amplifying the V4V5 region of the 16S rRNA gene with forward primer (3′-TGYCAGCCGCCGCGGTAA-5′) and reverse primer (3′-YCCGGCGTTGAVTCCAATT-5′). Paired-end reads were primer-cut, quality filtered, denoised, merged, de-chimerized, and dereplication using DADA2 [31]. The bacterial and archaeal taxonomy information of the generated amplicon sequence variants (ASVs) were annotated based on the Silva database (version 138.1).

### 2.3. Co-Occurrence Network Construction

Bacterial and archaeal amplicon sequence variants (ASVs) with a total average abundance greater than 0.05% were selected for bacterial and archaeal co-occurrence network construction, respectively. The correlation coefficients between individual ASVs were calculated based on the ‘trans_network’ function of the ‘microeco’ package in R (version 4.1.2) [32]. ASVs with correlation coefficients greater than 0.6 and significance less than 0.05 were screened for network construction and visualization. Network attributes were calculated using the ‘cal_network_attr’ function. Based on the among-module connectivities (Pi) and within-module connectivities (Zi) values calculated in ‘get_node_table’ function, the topological function of each node was estimated. Each sample subnetwork property was extracted based on the ‘subnet_property’ function, and the correlations between them and the environmental factors were calculated using the ‘cal_cor function’. Visualization of the co-occurrence networks was performed using Gephi (version 0.10.1).

### 2.4. Statistical Analysis

All statistical analyses and data visualization in this study were performed in R. Alpha diversity differences among groups were calculated using the cal_diff function based on the Kruskal–Wallis test in the ‘microeco’ package. Principal coordinate analysis (PCoA) based on the Bray–Curtis distance, which was performed using the ‘vegan’ package, and permutational multivariate analysis of variance (PERMANOVA) was conducted to assess the effect of sampling position on the microbial community. Microbial community assembly processes were quantified following the null model framework of Stegen et al. [23], utilizing two key metrics: βNTI and RC_Bray_ indices. These were computed using the ‘cal_ses_betamntd’ and ‘cal_rcbray’ functions in the ‘microeco’ package, respectively. βNTI > 2 and βNTI < −2 represent heterogeneous selection and homogeneous selection processes, respectively, and both further represent deterministic processes. In addition, |βNTI| < 2 and RC_bray_ > 0.95 were regarded as the influence of Dispersal Limitation and Drift; |βNTI| < 2 and RCbray < −0.95 were regarded as the influence of Homogenizing Dispersal; |βNTI| < 2 and |RCbray| < 0.95 were regarded as the influence of Drift acting alone. Correlations between environmental factors and bacterial and archaeal communities were tested using the Mantel test. The functional annotation of prokaryotic taxa (FAPROTAX version 1.2.6) was conducted to assess the ecological functions of the bacterial and archaeal community [33].

## 3. Results

### 3.1. Microbial Community Composition

**Figure 2:**
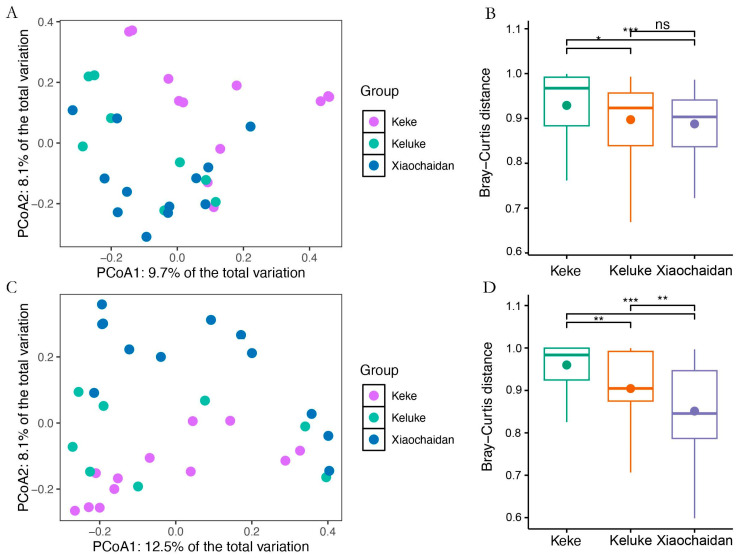
Differences in microbial community composition. (**A**) PCoA of bacterial communities based on Bray–Curtis distances. (**C**) PCoA of bacterial communities based on Bray–Curtis distances. The effects of sampling locations on bacterial and archaeal community composition were calculated using multinomial permutation ANOVA (* *p* < 0.05; ** *p* < 0.01; *** *p* < 0.001; ns represents no significant difference); differences in bacterial (**B**) and archaeal (**D**) community composition between sampling sites within groups.

A total of 1,501,821 high-quality bacterial sequences and 1,205,248 archaeal sequences were detected in the soils of *P. australis* communities around the three saline lakes. These sequences were annotated into 28,981 bacterial ASVs and 10,185 archaeal ASVs. The flattening species rarefaction curve indicated that the sequencing depth covered the vast majority of bacterial and archaeal species (Figure S1). The results of PCoA-based Bray–Curtis distance and PERMANOVA showed that sampling position significantly affected the composition of bacterial communities and archaeal communities (Figure 2A,C; *p* < 0.05). Subsequent post hoc analyses showed significant differences in soil bacterial and archaeal communities between the two surrounding the three saline lakes (*p* < 0.05). Notably, the community heterogeneity varied substantially across the sites. The bacterial and archaeal communities exhibited the greatest within-group variability at Keke Saline Lake, while showing the highest homogeneity at Xiaochadan (Figure 2B,D). The alpha diversity metrics remained largely consistent across locations, with the exception of archaeal Chao1 richness, which showed significant variation among the three lakes (Table S2).

### 3.2. Effect of Salinity on Microbial Community

**Figure 3:**
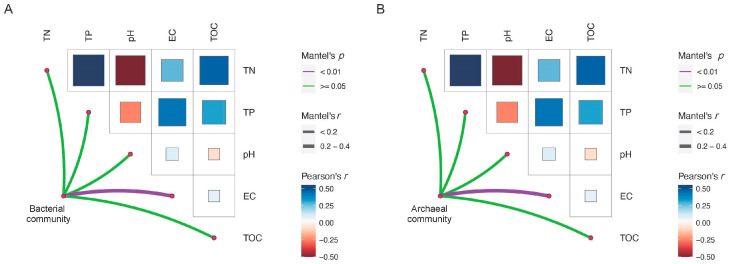
Mantel analysis revealing the significant correlation between environmental factors and bacterial community composition (**A**), and archaeal community composition (**B**). The color and size of the squares are associated with Pearson correlation coefficient of two environmental factors, and the color and size of edges is associated with Mantel analysis’ *p* and r values.

**Figure 4:**
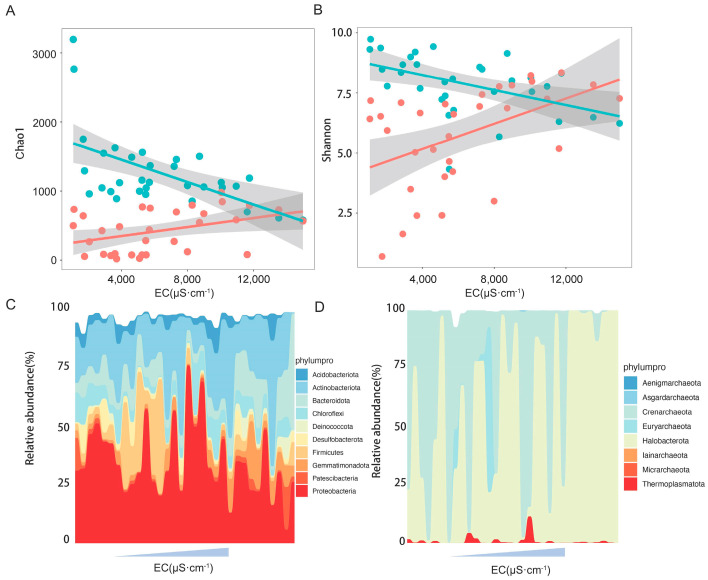
Effect of salinity on microbial communities. Regression analysis between Chao1 and electric conductivity (**A**), between Shannon and electric conductivity (**B**); blue and red colors represent bacterial and archaeal communities, respectively, while solid lines indicate a significant correlation between diversity and electric conductivity. Wave charts represent the dynamics of bacterial community (**C**) and archaeal community (**D**) phylum composition with increasing salt content, respectively.

Mantel analysis showed that soil electric conductivity was the key environmental factor influencing the community composition of bacteria and archaea (Figure 3). Shifts in alpha diversity and phylum composition of the microbial community with soil salinity were further investigated. The regression analysis results showed that the Chao1 and Shannon indices of bacterial communities were significantly negatively correlated with soil electric conductivity (Figure 4A,B), while those of the archaeal communities were significantly positively correlated with soil electric conductivity (Figure 4A,B). In terms of the bacterial and archaeal community composition at phylum level, Proteobacteria, Actinobacteriota, Firmicutes, Bacteroidota, Chloroflexi, and Gemmatimonadota were the dominant bacterial phylum, with a relative abundance representing over 87.0% of all bacterial sequences, and the predominant archaeal phylum Halobacterota and Crenarchaeota together occupying 98.5% of the total archaeal sequences. Significant turnover in bacterial phylum composition occurred with increasing soil salt concentration (Figure 4C). Among the dominant bacterial phyla, the relative abundance of Gemmatimonadota, Deinococcota, and Bacteroidota increased with increasing EC, whereas increasing EC significantly decreased the relative amount of Proteobacteria (Figure S2). In contrast, archaeal phyla demonstrated remarkable salinity tolerance, with none of the eight detected phyla showing significant abundance correlations with EC (Figure 4D and Figure S3).

### 3.3. Microbial Community Assembly Process

The null model analysis revealed that stochastic processes predominantly governed the assembly of both bacterial and archaeal communities in *P. australis* soil, with drift emerging as the dominant mechanism (accounting for 55.2% of bacterial and 61.1% of archaeal community assembly; Figure S4). Moreover, although deterministic processes contributed less to microbial community assembly, the variable selection was a major factor contributing to the deterministic process. In the case of the different saline lakes, the ecological processes involved in the assembly of soil bacterial and archaeal communities in *P. australis* communities were not the same. Stochastic processes were prevalent in bacterial communities and archaea across all sample sites, where the primary factors driving stochastic processes were drift and dispersal limitations, barring the archaeal community of Keke. Notably, with the exception of the bacterial community in Keluke and the archaeal community in Xiaochaidan, the determining processes for the bacterial and archaeal communities in the soils of the other sampling areas were mainly variable selection, which is related to environmental selection pressures.

### 3.4. Ecological Function of Microbial Community

**Figure 5:**
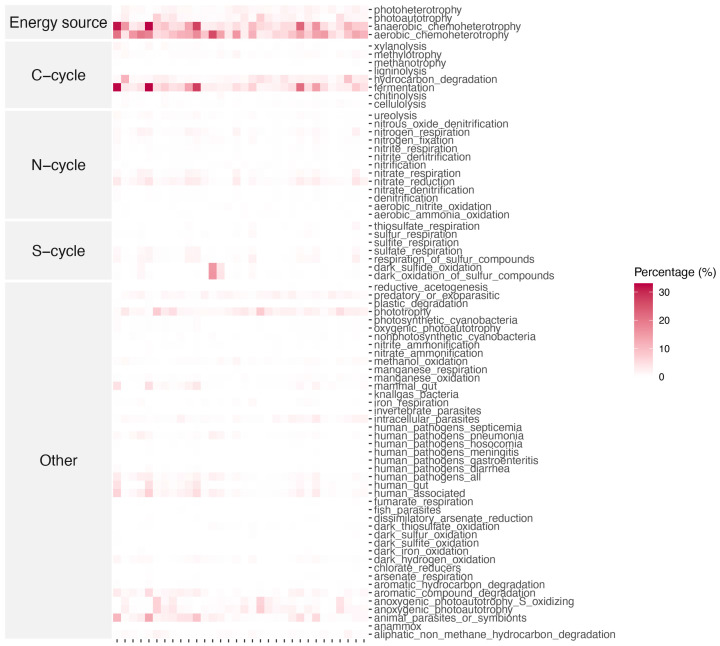
Bacterial community ecological function predicted by FAPROTAX according to the 16SrRNA gene sequencing data.

FAPROTAX-based prediction of microbial function showed that anaerobic and aerobic chemoheterotrophy capacities were the dominant forms of energy utilization in bacterial communities (Figure 5), whereas archaeal communities were predicted to be more biased towards energy production through aerobic metabolism (Figure S5). In terms of carbon cycling, bacterial communities were involved in C cycling, mainly through hydrocarbon degradation and fermentation pathways, while archaea played a key role in methane metabolism. In stark contrast, the archaeal community placed more capacity into the N cycle, especially nitrification and aerobic ammonia oxidation, whereas the bacterial community showed the exact opposite of the archaeal community in terms of nitrogen cycling processes, with a stronger nitrate reduction and nitrate respiration capacity. Notably, bacterial communities also played a key role in the cycling of sulfur. Correlation analysis revealed that EC had significant effects on the ecological function of bacterial communities. In contrast, the archaeal community function was more stable (Figures S6 and S7).

### 3.5. Co-Occurrence Network of Microbial Community

**Figure 6:**
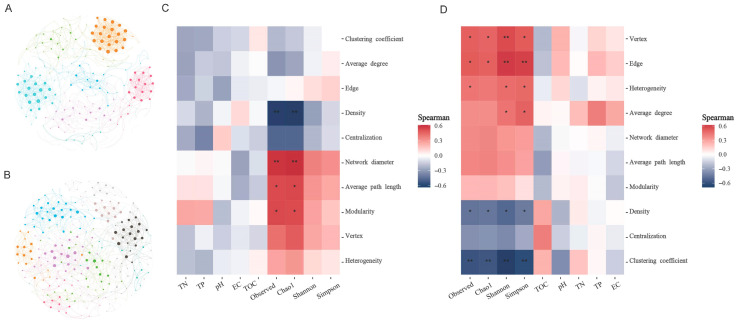
Co-occurrence networks of microbial community and the key factors affecting network topological properties. (**A**,**B**) represent the co-occurrence networks of bacterial community and archaeal community, respectively. Each node represents ASVs with a total average abundance greater than 0.05%, and the edges between each two nodes represent a strong significant correlation (Spearman > 0.6, and *p* < 0.05). The size of the nodes is proportional to the number of degrees per node. Heatmap showing key factors affecting bacterial (**C**) and archaeal (**D**) network attributes. * and ** represent *p* < 0.05 and *p* < 0.01, respectively, and red represents positive correlation and blue represents negative correlation.

Co-occurrence network analysis was performed to examine potential interaction patterns within bacterial and archaeal communities inhabiting P. australis soils adjacent to saline lakes (Figure 6). Nodes and edges were captured in the co-occurrence networks based on the significance (*p* < 0.05 and strong correlation (|*r*| > 0.06); the topological properties of co-occurrence networks are listed in Table S3). The average path lengths and average clustering coefficients of the bacterial and archaeal co-occurrence networks were smaller than those of the similarly sized Erdos–Renyi stochastic networks, suggesting that these networks had a ‘small-world’ character. Network interactions were predominantly cooperative, with bacterial networks showing 99.54% positive correlations and archaeal networks displaying exclusively positive interactions (100%). More nodes and edges were observed in the archaeal co-occurrence network than in the bacterial co-occurrence network, but the bacterial co-occurrence network showed higher levels of clustering coefficient, average degree, and density, indicating that bacterial networks were more tightly connected. The heatmap of the correlation between the topological properties of the subnetwork and the environmental factors showed that while environmental factors such as electric conductivity did not have a significant effect on the topological properties of the subnetwork, alpha diversity was a key factor influencing the properties of the subnetwork (Figure 6).

Moreover, Zi-Pi analysis was conducted to investigate the keystone taxa maintaining co-occurrence network stability. According to the Zi-Pi classification principle, nodes were divided into four categories: peripherals (Zi < 2.5 and Pi < 0.62), connector (Zi < 2.5 and Pi > 0.62), module hubs (Zi > 2.5 and Pi < 0.62), and network hubs (Zi > 2.5 and Pi > 0.62), where the connector, module hubs, and network hubs were keystones maintaining co-occurrence network stability (Figure S8). The results showed that both the bacterial communities had only one connector (ASV 24121) and module hub (ASV 15915), while the archaeal communities also had only one connector (ASV 10915) and module hub (ASV 4187). Counting the average relative abundance of keystones revealed that these keystone taxa were rare species (average relative abundance < 1%).

## 4. Discussion

### 4.1. Salinity Is a Key Environmental Factor Affecting Microbial Communities of Salt Marshes

As a widespread wetland plant globally, understanding the responses of the soil microbial composition, ecological functions, and community stability of *P. australis* communities to environmental disturbances is important for predicting the effects of global changes, including precipitation pattern changes and soil salinization [34,35]. Through Mantel analysis, we found that soil electrical conductivity was the predominant factor influencing the community composition of soil bacterial and archaeal communities, a conclusion that was also demonstrated by our previous study of soil bacteria in *P. australis* in the Yellow River Delta [36]. Salinity-induced shifts in microbial composition were evident through changes in alpha diversity (Chao1 and Shannon indices) and phylum-level distributions (Figure 4). Specifically, the bacterial diversity declined with increasing salinity, likely due to salt sensitivity in many taxa, which may enter dormancy or die under high-salt conditions. However, archaeal diversity was promoted by elevated soil salt concentrations, which have also been reported in lake sediments on the Tibetan Plateau [37]. This differential response reflects fundamental physiological differences: archaea possess unique adaptations such as ether-linked isoprenoid membrane lipids (glycerol-1-phosphate backbone), specialized motility structures, and distinct metabolic pathways, enabling them to thrive in extreme environments [38,39]. In addition, salinity-driven reductions in bacterial diversity may alleviate competitive pressure on archaea, facilitating their proliferation [40]. These findings underscore the complex interplay between salinity stress and microbial community dynamics in wetland ecosystems facing environmental change.

In terms of microbial composition, consistent with a previous study of the soil bacterial community in *P. australis* in the Yellow River Delta, the most dominant bacterial phylum was Proteobacteria, which have various energy acquisition methods, strong environmental adaptability, and rapid growth capacity [41,42]. However, while previous coastal studies reported increasing Proteobacteria abundance with salinity [14,32], we observed an opposite trend in these saline lake ecosystems. Contrastingly, the relative abundance of Proteobacteria increased with increasing soil salt concentration across various coastal ecosystems, but it presented a decreasing trend with increasing salt concentration in the current study [14,32]. This discrepancy suggests that beyond certain salinity thresholds (>8000 μS·cm^−1^), even adaptable Proteobacteria may be displaced by more halotolerant phyla such as Gemmatimonadota, Deinococcota, and Bacteroidota [6,43,44]. In contrast, archaeal communities demonstrated greater compositional stability, with Halobacterota and Crenarchaeota maintaining their dominance regardless of salinity fluctuations. The extreme halophily of Halobacterota enabled their persistence in high-salinity conditions, while salt-sensitive Crenarchaeota showed significant reductions in abundance only at extreme conductivities [45,46]. These results suggest that salinization plays a more crucial role in shaping bacterial phylum composition than archaeal phylum composition. These findings collectively indicate that while salinization exerts a strong selective pressure on bacterial phylum composition, archaeal communities maintain greater structural integrity across salinity gradients, highlighting fundamental differences in their environmental sensitivity and adaptive strategies.

The dynamics in the relative abundance of microorganisms as functioning agents inevitably alter the ecological functions of microbial communities. In the oxygen-limited environment characteristic of saline marsh soils, bacterial communities demonstrated remarkable metabolic flexibility, employing both anaerobic and aerobic chemoheterotrophic pathways for energy acquisition [47]. This adaptive capacity extended to nitrogen cycling, where bacteria mediated key processes including denitrification and nitrification [6]. Contrary to the conventional understanding of wetland archaeal ecology, our findings revealed a predominance of aerobic nitrogen-cycling pathways (nitrification and ammonia oxidation) over methanogenesis, which was restricted to isolated microhabitats [48]. This challenges the traditional paradigm of archaea primarily driving anaerobic methane production in wetland systems, instead aligning with emerging evidence of their crucial role in aerobic nitrogen transformations [49,50]. Salinity emerged as a critical regulator of microbial functionality, with elevated electrical conductivity constraining both bacterial and archaeal metabolic performance. This functional inhibition likely reflects the metabolic cost of maintaining osmoprotection mechanisms in hypersaline environments, diverting energy from other physiological processes. Overall, soil electric conductivity had a significant effect on the composition of bacterial and archaeal communities, the relative abundance of specific taxa, and microbial ecological functions.

### 4.2. Stochastic Process Driving Microbial Community Assembly

Currently, investigating microbial community assembly mechanisms represents a central challenge in microbial ecology, where both deterministic and stochastic processes collectively shape community structure [21]. Our investigation of bacterial and archaeal communities in *P. australis* soils across three Qinghai-Tibetan Plateau saline lakes revealed significant compositional differences (Table 1), reflecting a distinct ecological niche partitioning among lakes [51]. These findings align with established biogeographic patterns demonstrating distance–decay relationships in microbial communities, even at small spatial scales [35,52], driven by environmental filtering, dispersal limitations, and community complexity [53,54]. For these reasons, we evaluated the mechanisms of bacterial and archaeal community assembly in soil based on the null model, and the results showed that stochastic processes dominated both bacterial and archaeal communities. This result, in accordance with our previous research, revealed that stochastic assembly governed the bacterial assembly in the salt marsh ecosystems and archaeal assembly in mangrove sediments [19,24]. Based on a three-stage conceptual model of community assembly and an ecological simulation model, Dini-Andreote et al. [22] proposed that soil organic matter is a key predictor of the degree of influence of deterministic processes. Indeed, the more elementary stage of succession and the low soil organic matter content of the *P. australis* communities, both in the salt marsh wetland and around the saline lake, also dictated that the stochastic process became the main factor driving soil microbial assembly.

In the current study, the analysis of the stochastic process of bacterial and archaeal communities based on βNTI analysis showed that drift, homogeneous selection, and dispersal limitation were in decreasing order of importance (Figure 3). This finding challenges conventional microbial ecology paradigms, where drift is typically considered a secondary assembly force [23,55]. The prominence of drift in our system likely stems from two key factors: (1) exceptionally low microbial migration rates (bacteria: m = 0.0037; archaea: m = 0.0016), as evidenced by neutral modeling; and (2) small effective population sizes under saline stress conditions [56]. Homogeneous selection suggests that similar biotic and abiotic factors drive similar microbial community compositions between sites, whereas the dominant role of homogeneous selection in the present study may be the result of homogenized *P. australis* habitats [24,55]. As discussed earlier, the community similarity was greatest in the region of least distance, reflecting the fact of the formation of microbial communities well suited to specific environmental and biological conditions in one or adjacent regions where environmental conditions are fairly similar [21]. In other studies, dispersal limitation was found to be the predominant mechanism driving bacterial community assembly, and its high contribution to microbial assembly, in the current study, obviously reflected the separation of the different saline lakes [57].

### 4.3. Alpha Diversity Links to Microbial Co-Occurrence Network Complexity

Current ecological research emphasizes the understanding of how global change factors influence microbial community stability and the mechanisms governing micro-ecological resilience [27]. In this study, our results showed that alpha diversity of bacterial and archaeal communities had significant effects on co-occurrence network complexity. Specifically, as the alpha diversity increased, the number of nodes, connectivity, and modularity of the network increased, which improved the complexity of the network [28]. This finding was consistent with previous findings that microbial community diversity enhanced ecosystem complexity, and that a higher complexity responded to higher network stability [28,58]. It is worth noting that although electric conductivity did not have a significant effect on the network properties, it cannot be ignored that it inhibited and promoted alpha diversity in the bacterial and fungal communities, respectively. This means that increases in electric conductivity may modulate the complexity of microbial co-occurrence networks by affecting microbial diversity, but this cascading effect needs to be further investigated. Furthermore, subsequent studies should expand geographic sampling to validate our findings and employ metagenomic approaches to elucidate microbial interaction mechanisms.

## 5. Conclusions

Our study revealed the differences in diversity, composition, ecological function, co-occurrence network, and assembly process of the soil bacterial and archaeal communities of *Phragmites australis* salt marshes of the Qaidam Basin on the Qinghai-Tibetan Plateau. The main assembly mechanisms of bacterial and archaeal communities were stochastic processes, in which drift, homogeneous selection, and dispersal limitation were of decreasing importance. Soil bacterial and archaeal community composition was significantly correlated with soil electric conductivity, with the alpha diversity of the bacterial community showing a decreasing trend with increasing electric conductivity, while the alpha diversity of archaea increased with increasing electric conductivity. At the bacterial phylum level, the relative abundance of Proteobacteria was gradually replaced by the salt-resistant Gemmatimonadota, Deinococcota, and Bacteroidota as the electric conduction increased. Correspondingly, the ecological functions of bacterial and archaeal communities were significantly affected by soil electric conductivity. Co-occurrence network analysis showed that, although soil electric conductivity did not significantly affect the complexity of the microbial co-occurrence network, the alpha-diversity of the microbial community affected by it showed a significant correlation with network complexity. Our study also emphasizes that rare species play a crucial role in maintaining co-occurrence networks in plateau salt marshes.

## Figures and Tables

**Table 1:** Assembly mechanisms of bacterial and archaeal communities.

	Keke		Keluke		Xiaochaidan	
	Bacteria	Archaea	Bacteria	Archaea	Bacteria	Archaea
Variable Selection	36.36	24.24	0	7.14	16.67	0
Homogeneous Selection	0	0	7.14	0	1.51	0
Dispersal Limitation	16.67	3.03	21.43	21.43	19.70	27.27
Homogeneous Dispersal	12.12	15.15	7.14	14.29	1.52	0
Drift	34.85	57.58	64.29	57.14	60.61	72.73

## Data Availability

The raw sequencing data supporting this study are publicly available in the NCBI BioProject database under accession numbers PRJNA1127080 and PRJNA1127086. The soil data of physical and chemical properties can be found in Table S1 in the supplement file.

## Supplementary Materials

The following supporting information can be downloaded at https://www.mdpi.com/article/10.3390/microorganisms13061253/s1, Figure S1: Rarefaction Curve showing the trends in alpha of bacterial (A) and archaeal communities (B) with the depth of sequencing; Figure S2: Regression analysis between dominant bacterial phylum and EC, red line represents significant correlation; Figure S3: Regression analysis between dominant archaeal phylum and EC, red line represents significant correlation; Figure S4: Microbial community assembly mechanisms. A and B represent the proportion of deterministic and stochastic processes to bacterial and archaeal community assembly, respectively; Figure S5: Archaeal community ecological function predicted by FAPROTAX according to the amplicon sequencing data; Figure S6: Heatmap showing the correlation between soil physicochemcial properties and bacterial community ecological functions; Figure S7: Heatmap showing the correlation between soil physicochemcial properties and archaeal community ecological functions; Figure S8: Zi-Pi analysis of node of co-occurrence network. A and B excluded the keystone bacterial and archaeal taxa of co-occurrence network, respectively; Table S1: Location of soil sampling and physical and chemical properties of soil; Table S2: Alpha diversity of microbial community; Table S3: The attribute of co-occurrence network.
