# Supplementary material for: Salinity Effect on Soil Bacterial and Archaeal Diversity and Assembly in Phragmites australis Salt Marshes in the Qaidam Basin, China
Source: Microorganisms. 2025 May 29;13(6):1253. doi: 10.3390/microorganisms13061253 (PMC12195177; doi:10.3390/microorganisms13061253)
Supplement: Supplementary file 1 [file microorganisms-13-01253-s001.zip › microorganisms-3639374-supplementary.pdf]

Supplementary files

Table S1 Location of soil sampling and physical and chemical properties of soil

ID	TN (mg/kg)	TP (mg/kg)	pH	EC (μs/cm)	TOC (mg/g)	Longitude (E)	Latitude (N)	Altitude (m)
YH03	52	369.8	8.33	5090	19.69	98.267542	36.967571	2888.28
YH04	60	300.58	8.55	5264.67	22.3	98.267523	36.967599	2889.42
YH05	12	256.58	8.66	1076.67	8.77	98.267761	36.968196	2888.2
YH08	37	280.59	8.03	1120	9.01	98.266110	36.969981	2898.71
YH09	39	308.62	8.08	3699.67	2.7	98.265713	36.970025	2899.78
YH10	121	383.74	8.82	14990	18.69	98.265403	36.970835	2902.85
YH11	56	282.67	7.74	11613.33	3.18	98.264518	36.971914	2888.26
YH13	61	286.02	8.2	10042.33	13.81	98.262930	36.973256	2891.6
YH14	97	294.11	7	5239.67	2.62	98.262776	36.974494	2899.53
YH15	78	293.2	7.28	3605.67	6.13	98.261747	36.974270	2898.18
YH16	118	171.43	7.11	3351.33	6.42	98.260910	36.974674	2897.2
YH17	85	322.26	7.81	2913.67	13.5	98.260528	36.974919	2897.75
YH34	328	339.26	7.76	5737	39.7	96.901344	37.316139	2768.8
YH35	244	377.25	7.81	5686	21.3	96.899830	37.317133	2763.09
YH36	146	426.51	8.44	7322.33	4.86	96.903579	37.316525	2768.47
YH37	130	378.39	7.72	1678.67	3.18	96.904211	37.316562	2766.95
YH38	207	436.53	8.15	8279.33	8.62	96.904807	37.316678	2770.58
YH39	220	397.12	8.13	8723.33	17.44	96.906114	37.316072	2772.11
YH40	210	371.26	7.4	10103.67	7.85	96.906830	37.316011	2768.31
YH41	231	362.42	7.43	11750	15.3	96.907655	37.315595	2769.7
YH51	28	252.16	9.22	10946.67	2.02	95.410021	37.537160	3129.02
YH52	86	293.64	8.8	7994	13.57	95.410951	37.536749	3129.4
YH53	46	348.92	9.19	7187.33	2.63	95.411423	37.536435	3127.9
YH54	28	281.05	8.55	4617	16.1	95.412022	37.536153	3128.41
YH55	66	323.78	8.75	5466	16.57	95.412593	37.535963	3162.71
YH56	60	245.02	8.29	5491	14.63	95.413027	37.535896	3131.21
YH57	21	172.24	9.15	1759	11.1	95.413944	37.536931	3137.92
YH58	68	279.65	9.17	13526.67	0.92	95.408953	37.538132	3136.58
YH59	59	379.68	9.14	8986	22.3	95.407401	37.538570	3127.77
YH60	40	174.16	8.79	2816.67	11.65	95.406876	37.538574	3129.26
YH61	90	158.82	9.08	2046.33	1.17	95.406487	37.538746	3133.05
YH62	18	182.49	9.31	3881.33	0.66	95.405900	37.539039	3142.93

Table S2 Alpha diversity of microbial community

Group	Arc_Chao1	Arc_Shannon	Bac_Chao1	Bac_Shannon
Keke	332.23±349.91b	3.48±1.54a	1439.51±814.13a	5.65±0.81a
Keluke	688.04±136.93a	4.83±0.90a	1286.63±284.58a	5.50±0.85a
Xiaochaidan	333.89±242.88b	3.97±1.47a	1090.38±221.99a	5.25±0.91a

Table S3 The attribute of co-occurrence network

		Bacteria	Archaea
Empirical network	Node	226	268
	Edge	871	906
	Average_degree	7.7079646	6.76119403
	Average_path_length	3.50798933	3.7737
	Network_diameter	10	11
	Clustering_coefficient	0.71628136	0.49687
	Density	0.03425762	0.02532
	Heterogeneity	0.80416402	0.56835
	Centralization	0.0679646	0.03834
	Modularity	0.76216663	0.7806
Random network	Average_path_length	2.86	3.13
	Clustering_coefficient	0.028	0.024

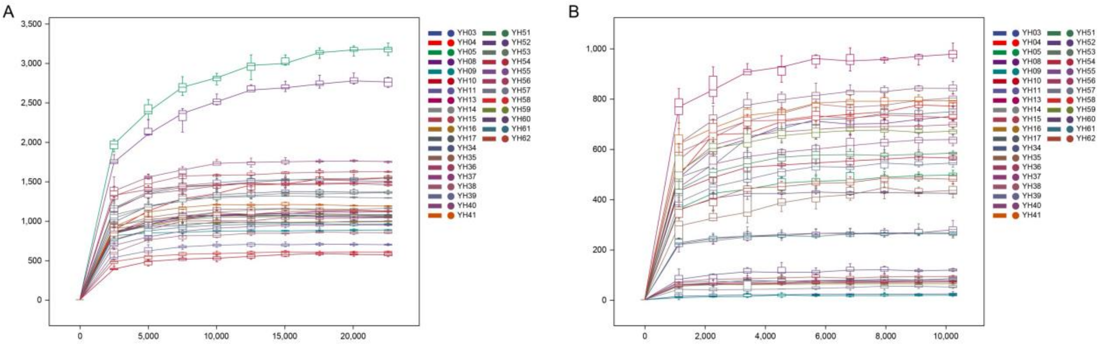

Figure S1 Rarefaction Curve showing the trends in alpha of bacterial (A) and archaeal communities(B) with the depth of sequencing.

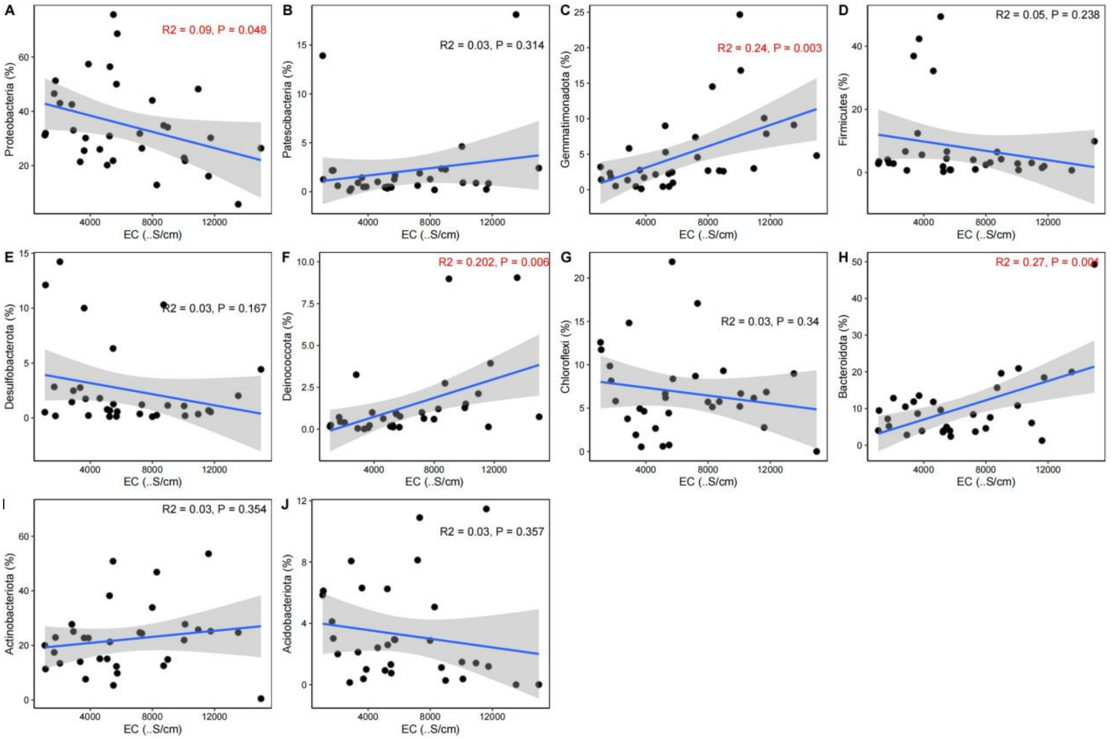

Figure S2 Regression analysis between dominant bacterial phylum and EC, red line represented the significant correlation.

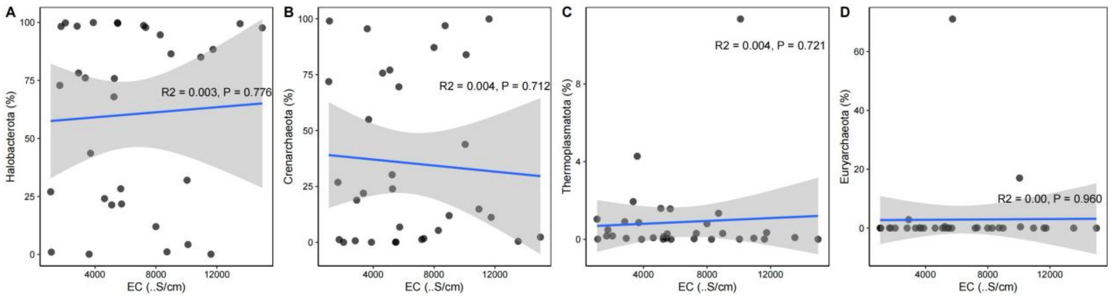

Figure S3 Regression analysis between dominant archaeal phylum and EC, red line represented the significant correlation.

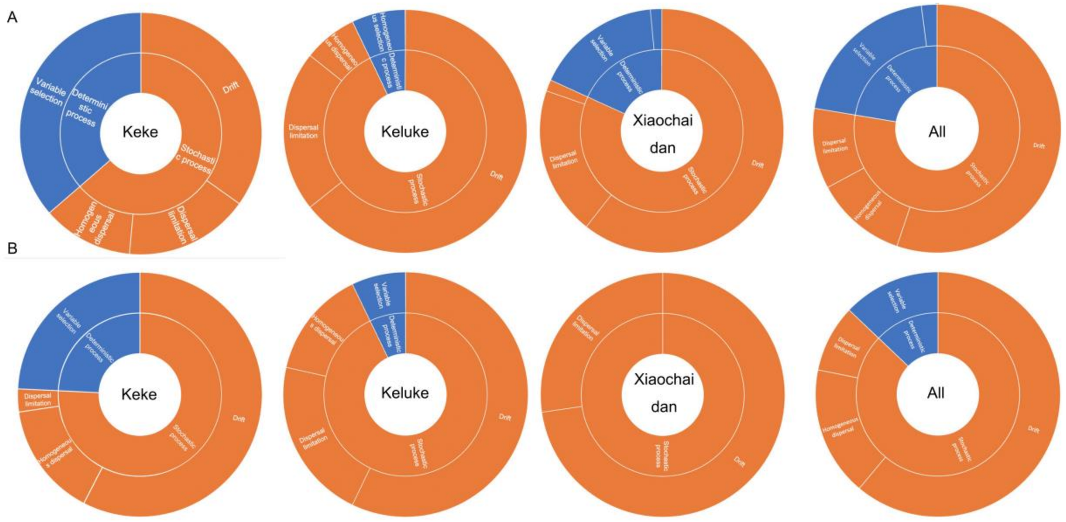

Figure S4 Microbial community assembly mechanisms. A and B represent the proportion of deterministic and stochastic processes to bacterial and archaeal community assembly, respectively.

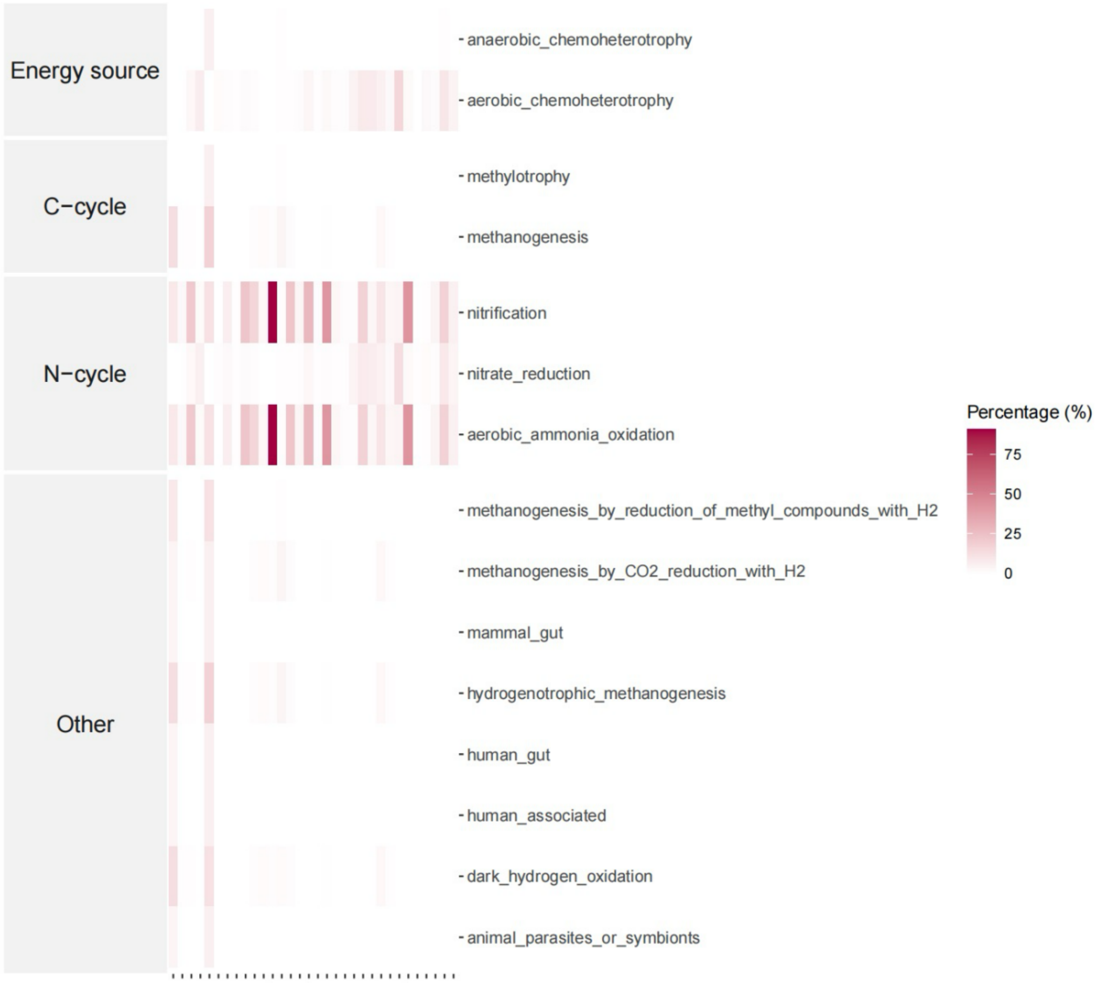

Figure S5 Archaeal community ecological function predicted by FAPROTAX according to the amplicon sequencing data.

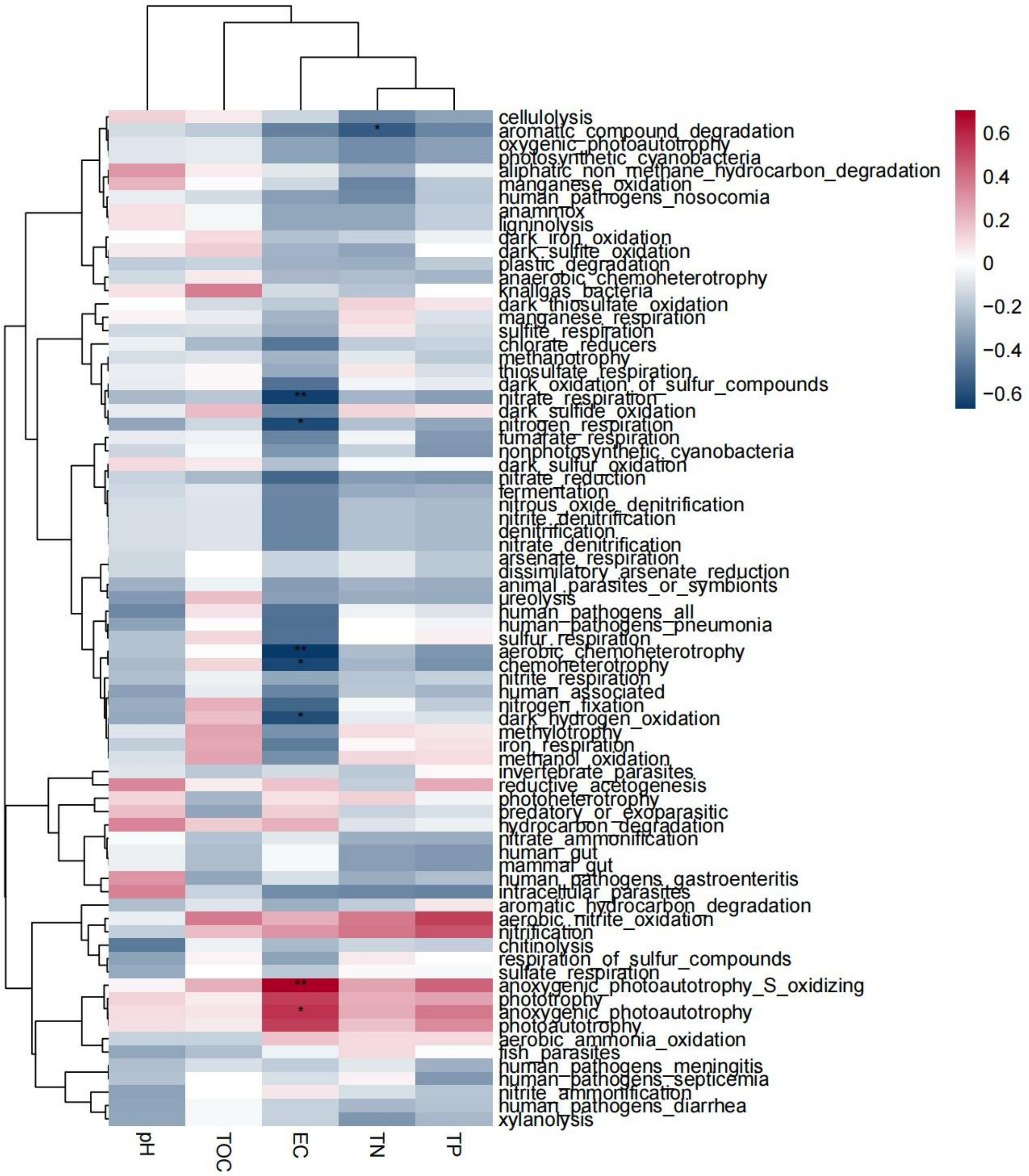

Figure S6 Heatmap showing the correlation between soil physicochemcial properties and bacterial community ecological functions.

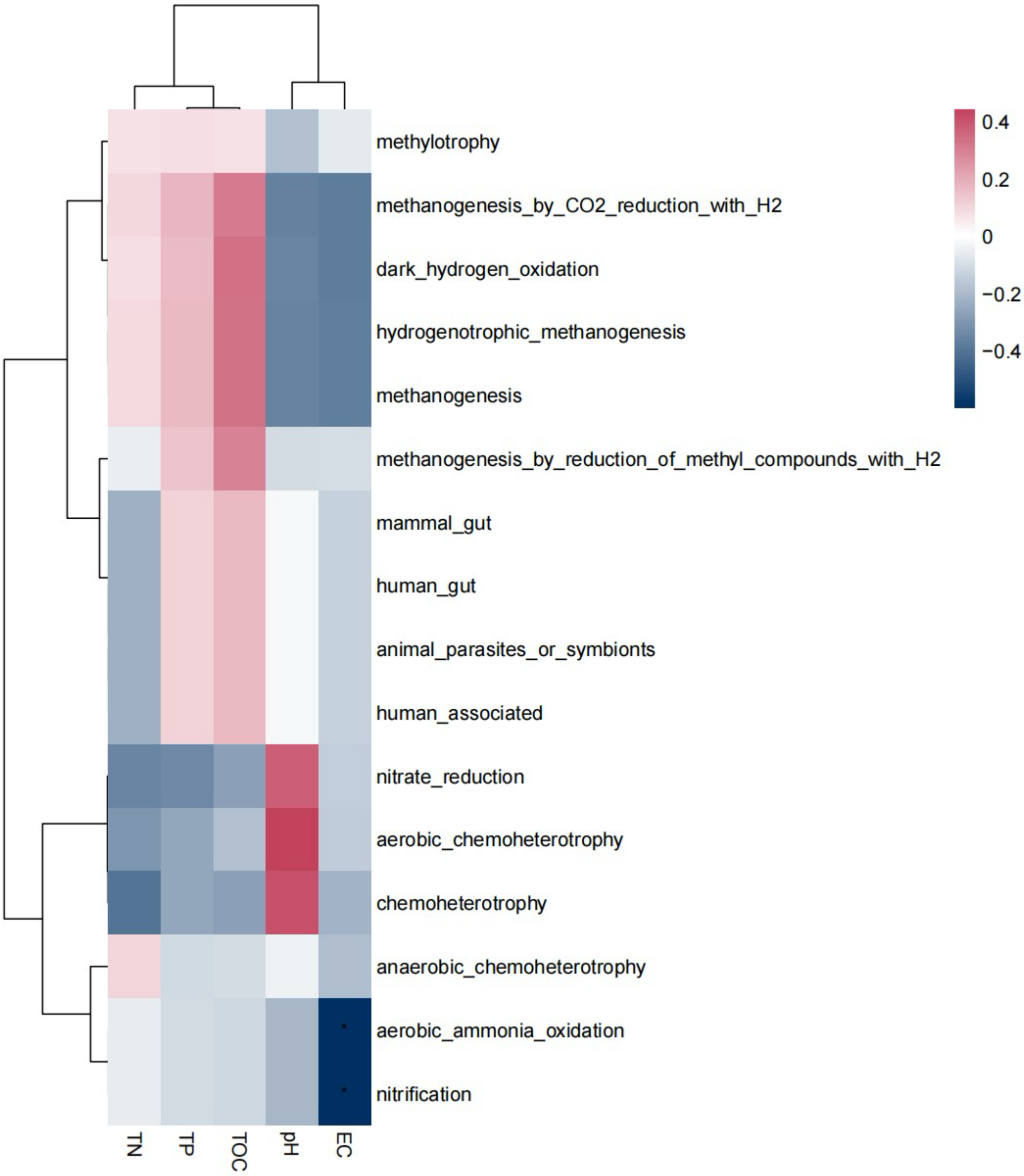

Figure S7 Heatmap showing the correlation between soil physicochemcial properties and archaeal community ecological functions.

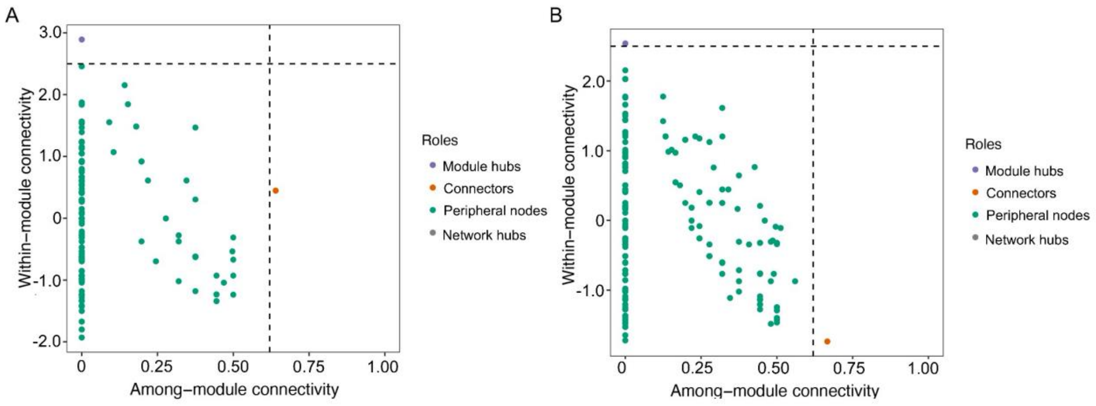

Figure S8 *Zi-Pi* analysis of node of co-occurrence network. A and B excluded the keystone bacterial and archaeal taxa of co-occurrence network, respectively.
